# Systemic Therapy of Metastatic Pancreatic Adenocarcinoma: Current Status, Challenges, and Opportunities

**DOI:** 10.3390/cancers14112588

**Published:** 2022-05-24

**Authors:** Sakti Chakrabarti, Mandana Kamgar, Amit Mahipal

**Affiliations:** 1 Medical College of Wisconsin, 8701 Watertown Plank Road, Milwaukee, WI 53226, USA; schakrabarti@mcw.edu (S.C.); mkamgar@mcw.edu (M.K.); 2 Division of Medical Oncology, Mayo Clinic, 200 First Street SW, Rochester, MN 55905, USA

**Keywords:** pancreatic ductal adenocarcinoma (PDAC), targeted therapy, next-generation sequencing, maintenance therapy

## Abstract

**Simple Summary:**

Pancreatic ductal adenocarcinoma (PDAC) is a common type of cancer originating from the pancreatic glands and is characterized by a rapidly progressive course and a dismal prognosis. Currently, chemotherapy is the cornerstone of treatment that has modest efficacy. Targeted treatment options are rapidly evolving with the growing understanding of pathobiological underpinnings of tumorigenesis, progression, and treatment resistance mechanisms. Combination strategies targeting multiple signaling pathways supporting tumor growth and propagation are active areas of contemporary research that will likely transform the treatment paradigm of pancreatic cancer.

**Abstract:**

Pancreatic ductal adenocarcinoma (PDAC) is an aggressive malignancy characterized by nonspecific presenting symptoms, lack of a screening test, rapidly progressive clinical course, and presentation with an advanced-stage disease in the majority of patients. PDAC is essentially a systemic disease irrespective of the initial stage, as most patients with non-metastatic PDAC undergoing curative-intent treatment eventually experience metastatic relapse. Currently, cytotoxic chemotherapy remains the cornerstone of treatment in patients with advanced disease. However, the current standard treatment with multiagent chemotherapy has modest efficacy and results in median overall survival (OS) of less than a year and a 5-year OS of about 10%. The pathobiology of PDAC poses many challenges, including a unique tumor microenvironment interfering with drug delivery, intratumoral heterogeneity, and a strongly immunosuppressive microenvironment that supports cancer growth. Recent research is exploring a wide range of novel therapeutic targets, including genomic alterations, tumor microenvironment, and tumor metabolism. The rapid evolution of tumor genome sequencing technologies paves the way for personalized, targeted therapies. The present review summarizes the current chemotherapeutic treatment paradigm of advanced PDAC and discusses the evolving novel targets that are being investigated in a myriad of clinical trials.

## 1. Introduction

Pancreatic ductal adenocarcinoma (PDAC) is a lethal malignancy that originates in the exocrine pancreatic glands. PDAC is the second-most frequent malignancy arising from the digestive tract, with an estimated annual incidence of 60,430 in the United States of America (USA) [1] and 340,000 worldwide [2]. PDAC has several unique features, including the rapidly progressive nature of the tumor, the proximity of the pancreas to important vascular structures, often precluding curative resection, absence of typical presenting signs or symptoms that make the diagnosis and treatment difficult [3]. PDAC is the fourth leading cause of cancer-related death in the USA [1], and the aggressive biology of PDAC is reflected in its poor 5-year relative survival rate of 10.8% [4]. It is important to note that the incidence of PDAC is increasing at such a pace that PDAC is projected to be the second leading cause of cancer mortality by the year 2030 [5]. Furthermore, approximately 80% of patients with localized PDAC harbor micrometastatic disease, evidenced by a high rate of cancer relapse in patients with localized PDAC receiving curative-intent treatment [6]. These data underscore the urgent need for effective systemic therapies for PDAC.

Chemotherapy is currently the cornerstone of systemic therapy for patients with metastatic PDAC (mPDAC), which results in median overall survival (mOS) of less than a year [7,8]. The evolution of systemic therapy for pancreatic cancer has been rather gradual, from gemcitabine monotherapy [9,10,11,12] to the current standard of combination chemotherapy with triplet [7] or doublet agents [8]. However, multidrug chemotherapy regimens have been associated with modest survival gain and significant toxicities limiting their utility. Advancements in the understanding of tumor biology, accelerated by the rapid evolution of genomic sequencing technologies, have led to newer therapies, including immunotherapy and targeted therapies, that hold significant promise. The present review provides a detailed account of the current treatment strategies and developing therapeutic paradigms that have the potential to transform the treatment of advanced pancreatic cancer dramatically.

## 2. Advanced PDAC: Current Standard of Care

Advanced PDAC is incurable, and hence the treatment goals include controlling the tumor growth to prolong life, controlling symptoms, and maintaining the quality of life. The survival benefit of systemic chemotherapy over best supportive care has long been established in patients with mPDAC [13,14]. The current standard treatment consists of multiagent chemotherapy regimens in patients with good Eastern Cooperative Oncology Group (ECOG) performance status (PS), although the toxicity limits the utility of such regimens. Consequently, maintenance therapy following an initial period of induction therapy is being investigated. Immunotherapy has been accepted as a standard treatment in a minority of patients with deficient mismatch repair (dMMR) tumors.

### 2.1. First-Line Chemotherapy

Gemcitabine monotherapy was established as the standard of care for patients with mPDAC in the 1990s based on a randomized trial. In this trial, treatment-naïve patients with mPDAC received single-agent gemcitabine vs. 5-FU. The mOS improved modestly with gemcitabine (5.65 months vs. 4.41 months with 5-FU, *p* = 0.0025) [10]. Although a subsequent study with a combination of gemcitabine and erlotinib, an epidermal growth factor receptor (EGFR) inhibitor, showed a slightly better mOS compared to gemcitabine monotherapy (6.2 months vs. 5.9 months) [15], single-agent gemcitabine remained the standard first-line chemotherapy until 2011 owing to significant toxicities associated with the gemcitabine-erlotinib combination for a relatively small clinical benefit. Gemcitabine monotherapy is currently reserved for patients with an ECOG PS of 2 or higher or comorbidities precluding multiagent chemotherapy regimens. Table 1 summarizes the first-line studies.

In treatment-naïve patients of mPDAC with good ECOG PS, two multiagent regimens, FOLFIRONOX (5-FU, irinotecan, and oxaliplatin) and gemcitabine/nab-paclitaxel (GnP), are currently the preferred regimens. PRODIGE 4, a phase III randomized trial, compared FOLFIRINOX with gemcitabine monotherapy in treatment-naïve patients with mPDAC and reported a significant survival benefit with FOLFIRINOX among 342 treatment-naïve patients (mOS: 11.1 vs. 6.8 months, 95% CI, 0.45–0.73; *p* < 0.001) [7]. The overall response rate (ORR) and median progression-free survival (mPFS) also favored FOLFIRINOX (ORR: 31.6% vs. 9.4%, mPFS: 6.4 vs. 3.3 months). FOLFIRINOX, however, was associated with significantly higher rate of grade 3 or 4 neutropenia (45.7% vs. 21%), febrile neutropenia (5.4% vs. 1.2%), diarrhea (12.7% vs. 1.8%), and sensory neuropathy (9% vs. 0%) compared to gemcitabine. The role of GnP in the first-line setting was established by a phase III trial that randomly assigned 861 previously untreated patients to either GnP or gemcitabine [8]. The GnP regimen was associated with significantly improved mOS (8.5 vs. 6.7 months, HR 0.72, *p* < 0.001), mPFS (5.5 vs. 3.7 months, HR 0.69, *p* < 0.001), and ORR (23% vs. 7%, HR 3.19, *p* < 0.001). However, grade 3 or higher toxicities were more frequent with GnP (neutropenia 38% vs. 27%, peripheral neuropathy 17% vs. 1%, and diarrhea 6% vs. 1%). It should be noted that, unlike the PRODIGE4 study, this trial enrolled over 60% of patients from the USA with relatively less robust performance scores (Karnofsky performance score of 70 or more). In addition, there was no age limit for participation in this trial.

Concerns related to the toxicities with FOLFIRINOX triggered several subsequent studies with modified FOLFIRINOX regimens. A prospective, single-arm, phase II study evaluated a modified FOLFIRINOX regimen (oxaliplatin 85 mg/m^2^, irinotecan 135 mg/m^2^, 5-FU 300 mg/m^2^ bolus, followed by 2400 mg/m^2^ continuous infusion for 46 h every 14 days) in patients with metastatic and locally advanced PDAC (*n* = 31) [19]. The study reported a much lower incidence of Grade 3 or 4 neutropenia and diarrhea, 12.2% and 16.2%, respectively. Furthermore, the efficacy appeared to be comparable to the PRODIGE 4 study results with an mOS of 10.2 months (95% CI, 7.6 to 14.3), an mPFS of 6.1 months (95% CI, 5.1 to 8.3), and an ORR of 35.1%. Another retrospective study with metastatic and nonmetastatic PDAC (*n* = 60) patients evaluated a different version of modified FOLFIRINOX regimen that consisted of oxaliplatin 85 mg/m^2^, irinotecan 180 mg/m^2^, and 5-FU 2400 mg/m^2^ infusion over 46 h without a 5-FU bolus (*n* = 60) [20]. The mOS and mPFS were 9 months (95% CI, 7.1 to not estimable) and 8.5 months (95% CI, 3.7 to 11), respectively. The ORR was 30%, with the rate of grade 4 neutropenia, grade 3/4 diarrhea, and fatigue of 3%, 13%, and 13%, respectively. Subsequently, a large meta-analysis that included 1461 patients in 32 studies showed no difference in mOS, mPFS, and ORR between the standard FOLFIRINOX regimen utilized in the PRODIGE4 study and the modified FOLFIRINOX regimens [21]. Based on these studies, modified FOLFIRINOX regimens have been adopted widely in routine clinical practice.

A prospective randomized trial has not been completed to date comparing FOLFIRINOX with GnP in untreated patients with mPDAC. However, several retrospective studies have suggested similar efficacy and safety with both regimens [22,23]. A meta-analysis of 16 retrospective studies that included 3813 patients (1690 patients treated with FOLFIRINOX and 2123 patients treated with GnP) reported similar mOS (HR 0.99; 95% CI, 0.84–1.16; *p* = 0.9), mPFS (HR 0.88; 95% CI, 0.71–1.1; *p* = 0.26), and ORR (25% with GnP vs. 24% with FOLFIRINOX) with both regimens [24]. However, a median weighted OS difference favored FOLFIRINOX (mean difference: 1.15, 95% CI, 0.08–2.22, *p* = 0.03), and the toxicity profile was different, grade 3/4 neutropenia, nausea, and febrile neutropenia were higher with FOLFIRINOX, while neurotoxicity and anemia were higher with GnP. Two-phase III randomized trials are currently underway comparing modified FOLFIRINOX and GnP in treatment-naïve patients with mPDAC (NCT04229004 and NCT04469556).

A single-arm, open-label, phase 1b/2 study investigated the role of a triplet regimen (cisplatin, gemcitabine, and nab-paclitaxel) in 25 patients with previously untreated mPDAC and reported an ORR of 71%, a disease control rate (DCR) of 88%, mOS of 16.4 months (95% CI, 10.2–25.3), and mPFS of 10.1 (95% CI, 6.0–12.5) months [16]. Based on this study, a single-arm phase II open-label study (NCT03915444) with the triplet regimen in untreated mPDAC patients has fully accrued recently, the results of which are pending at this time.

An open-label, phase I/II study has recently evaluated the safety and efficacy of NALIRIFOX (liposomal irinotecan + 5-FU + leucovorin + oxaliplatin) in patients with mPDAC in the first-line setting [18]. Among the 32 patients who received the maximum tolerated dose (MTD) that consisted of liposomal irinotecan 50 mg/m^2^, oxaliplatin 60 mg/m^2^, 5-fluorouracil 2400 mg/m^2^, and leucovorin 400 mg/m^2^ every 2 weeks, the ORR, mPFS, and mOS were 34.4%, 9.2 months (95% CI, 7.69–11.96), and 12.6 months (95% CI, 8.74–18.69), respectively. In this study population, 22 of 32 had grade ≥3 treatment-related adverse events (TRAEs), including neutropenia (31.3%), febrile neutropenia (12.5%), and hypokalemia (12.5%). A phase III randomized study (NAPOLI 3) is currently underway, comparing NALIRIFOX to GnP in the first-line setting (NCT 04083235).

Germline mutations in the genes that are critical for the homologous recombination repair of DNA, including *BRCA1/2* and *PALB2* genes, are well-known predisposing factors for developing pancreatic cancer [25]. Approximately 5% to 9% of patients with PDAC harbor mutations in *BRCA1/2* or *PALB2* genes [25]. Previous studies have reported that mutations in *BRCA1/2* and *PALB2* genes are associated with increased sensitivity to platinum agents and synthetically lethal interaction with polyadenosine diphosphate-ribose polymerase (PARP) inhibitor (PARPi) [26]. Studies demonstrating a high response rate with cisplatin, gemcitabine, and a PARPi veliparib in patients with mPDAC [27,28] led to a randomized phase II trial to evaluate the efficacy of gemcitabine, cisplatin with or without veliparib in mPDAC patients with germline *BRCA/PALB2* mutations [17]. Although this study did not show improved efficacy with the addition of veliparib, the ORR, mPFS, and mOS were impressive with the cisplatin and gemcitabine combination, with an ORR of 65.2%, an mPFS of 9.7 months, and an mOS of 16.4 months. This study established cisplatin and gemcitabine combination as one of the first-line treatment options in PDAC patients harboring BRCA/PALB2 mutations.

GABRINOX, a prospective, single-arm phase II study, evaluated a sequential strategy in which 58 treatment-naïve patients with mPDAC received GnP (day 1, 8, and 15) followed by FOLFIRINOX (day 29 and 43) [29]. The regimen was well-tolerated, and the patients received a median of 4 (1–9) cycles in 8.5 months (0.5–19.8 months). The study reported an impressive ORR of 64.9% (95% CI, 51.1 to 77.1%), including 3.5% of CR, an mPFS of 10.5 months, and an mOS of 15.1 months. Of note, the incidence of grade 3 or higher peripheral neuropathy was remarkably low (5.2%).

### 2.2. Maintenance Therapy

Toxicities, especially neurotoxicities and cytopenias, are major barriers to the long-standing use of FOLFIRINOX or GnP in advanced pancreatic cancer patients. Consequently, several clinical trials have evaluated de-escalation after a period of induction therapy termed maintenance therapy. The FDA approval of maintenance treatment with a PARP inhibitor, olaparib, for adult mPDAC patients with deleterious germline BRCA mutations who have not progressed on the first-line platinum-based chemotherapy has brought the discussion around maintenance therapy in advanced pancreatic cancer to the forefront. The FDA approval of olaparib was based on the POLO trial [30], a phase III study in which 154 patients with mPDAC harboring germline BRCA 1 or 2 mutations remaining progression-free on the first-line platinum-containing regimen for at least 16 weeks were randomized to olaparib or placebo (92 patients to olaparib and 62 patients to placebo) [30]. The mPFS in the olaparib group was significantly longer than in the placebo group (7.4 months vs. 3.8 months; HR: 0.53; 95% CI, 0.35 to 0.82; *p* = 0.004). However, the final mOS was similar between the two arms- 19 months in the olaparib arm vs. 19.2 months in the placebo arm, *p* = 0.3487 [31]. Of note, the 36-month OS rate was higher in the olaparib arm (33.9% vs. 17.8%). The FDA approval of olaparib maintenance therapy based on the POLO trial data faced several critiques, including a comparison of olaparib maintenance with the placebo rather than more commonly practiced maintenance chemotherapy (for example, FOLFIRI [5-FU plus irinotecan] or single-agent 5-FU), and the 16 weeks of induction chemotherapy has been viewed as a rather short duration of induction therapy, and the lack of OS benefit. It is important to note that the study was not adequately powered to detect a difference in OS and 26% of patients in the placebo arm subsequently received a PARP inhibitor, potentially extending the OS in the placebo arm, although cross-over was not allowed in the trial. The other important consideration is that 40% of patients in the olaparib group experienced grade 3 or higher toxicities (most commonly anemia, fatigue, and decreased appetite), and toxicities led to discontinuation of olaparib in 5% of patients [30]. A similar study reported significant activity of another PARP inhibitor, rucaparib, as maintenance therapy in advanced PDAC patients with germline or somatic pathogenic variant mutation in *BRCA1*, *BRCA2*, or *PALB2* [32]. Eligible patients received a minimum of 16 weeks of platinum-based chemotherapy without any evidence of platinum resistance, followed by rucaparib 600 mg orally twice a day until progression. The PFS at 6 months (primary endpoint) was 59.5% (95% CI, 44.6 to 74.4), median PFS was 13.1 months (95% CI, 4.4 to 21.8), and median OS was 23.5 months (95% CI, 20 to 27).

The other maintenance therapy strategies include induction FOLFIRINOX followed by 5-FU [33,34] or capecitabine or FOLFIRI [33] or FOLFOX [33], induction GnP followed by gemcitabine maintenance [35], and maintenance sunitinib after first-line induction chemotherapy [36]. The optimal maintenance strategy, however, is not defined. The studies evaluating various maintenance treatment strategies are summarized in Table 2.

### 2.3. 2nd Line Chemotherapy

Approximately one-third to half of the patients with mPDAC progressing on first-line chemotherapy received second-line chemotherapy [37]. The prospective data supporting the benefit of second-line chemotherapy over best supportive care (BSC) are sparse. A phase III study by the German CONKO study group randomized patients with mPDAC progressing on gemcitabine to OFF (5-FU, folinic acid, and oxaliplatin) plus BSC or BSC alone. The patient group receiving OFF plus BSC had a significantly longer median survival from the initiation of second-line treatment (4.8 vs. 2.3 months) and also from the initiation of first-line gemcitabine (9 vs. 7.9 months, *p* = 0.031). Of note, a survival benefit with second-line treatment after progression on currently available multiagent first-line regimens (FOLFIRINOX or GA) is not supported by any prospective data.

The decision to pursue second-line therapy and the choice of therapy depend on a variety of factors, including performance status, residual toxicity from previous treatment, first-line therapy administered, comorbidities, presence of targetable genomic alterations, and patient preference.

The second-line chemotherapy regimens can be divided into two broad groups—(1) regimens for patients who progressed on FOLFIRINOX and (2) regimens for patients who progressed on first-line gemcitabine-based regimens (Table 3). After progression on FOLFIRINOX, treatment with GnP is feasible, as evaluated in two trials [38,39], although neuropathy could be an important barrier. Single-agent gemcitabine is a reasonable option in patients with significant residual neuropathy or inadequate performance status [40].

After progression on first-line gemcitabine, a wide variety of regimens are available, including liposomal irinotecan plus 5-FU/leucovorin combination [41], OFF (oxaliplatin, 5-FU/LV) [42], modified FOLFOX [43], modified FOLFIRINOX [44,45], FOLFIRI [46], XELOX [47], and docetaxel plus oxaliplatin [48] combination. Although none of these regimens is clearly superior to others, a meta-analysis that compared the efficacy of adding oxaliplatin vs. irinotecan formulations to a fluoropyrimidine after progression on first-line gemcitabine reported that the combination of a fluoropyrimidine plus irinotecan significantly improved both mPFS and mOS (HR: 0.7; 95% CI, 0.55–0.89), while oxaliplatin-based combinations modestly improved PFS but not the OS [49]. Table 3 summarizes important second-line studies in mPDAC.

## 3. Oligometastatic Disease

A subset of patients with mPDAC has limited metastatic disease, termed the oligometastatic disease, and may derive benefit from aggressive systemic therapy combined with resection of metastatic tumors. Although a well-agreed definition of oligometastatic pancreatic cancer does not exist, a proposed definition [50] includes the following criteria: (1) four or fewer hepatic or pulmonary metastatic lesions, (2) CA 19–9 of less than 1000 U/mol, (3) patients who responded to systemic chemotherapy. Approximately 8% of patients with mPDAC satisfy the criteria for oligometastatic disease [51]. The role of metastasectomy has not been well defined in mPDAC and should be restricted to select patients. The best candidates are patients who achieve prolonged radiologic and biochemical responses and have low-volume, lung-only metastases.

The optimum treatment protocol of oligometastatic mPDAC is not established. A systematic review and meta-analysis of six observational retrospective studies that included 2087 patients reported higher survival rates in patients who received chemotherapy followed by surgery vs. patients who received chemotherapy alone [52], with an mOS of 23 to 56 months after surgery compared to 11 to 16.4 months after chemotherapy alone. In this systematic review, FOLFIRINOX was found to be the most common chemotherapy regimen utilized. A single-center retrospective study (*n* = 85) reported an mOS of 12.3 months and a 5-year survival of 8.1% [53]. Another single-center retrospective study (*n* = 78) suggested that oligometastatic mPDAC with lung only metastasis has an indolent course, and the mOS was prolonged in patients who underwent surgery or stereotactic radiotherapy (67.5 months) vs. patients who received chemotherapy only (33.8 months) or observation (29.9 months) [54].

## 4. Therapies Targeting Genomic Alterations

The most common genomic alteration in PDAC is Kirsten rat sarcoma viral oncogene homolog (*KRAS*) mutation which is present in ~90% of tumors [55]. Point mutations at *G12* (98%), *G13* (1%), or *Q61* (1%) residues of *KRAS* lead to the constitutive activation of *RAS* and its downstream signaling pathways leading to tumor development and maintenance [55]. The direct targeting of *KRAS* or its downstream pathway is a major active area of research in PDAC.

### 4.1. Targeting KRAS

*KRAS G12C*, accounting for <2% of *KRAS* mutations in PDAC [56], can be targeted with covalent inhibitors that lock *KRAS G12C* in its inactive GDP bound form [56]. Adagrasib (MRTX849) is one such inhibitor with promising preliminary results among PDAC patients in the ongoing KRYSTAL-1 trial [57]. Among the 10 evaluable PDAC patients enrolled in the KRYSTAL-1 study, adagrasib 600 mg twice daily was associated with an ORR of 50%, disease control rate (DCR) of 100%, an mPFS of 6.6 months (95% CI, 1.0–9.7), and 50% of these patients have ongoing responses at the time of data analysis (median follow up 8.1 months) [57]. Sotorasib (AMG510), another drug with the same mechanism of action which is FDA approved for *KRAS G12C* mutated non-small cell lung cancer, showed modest, short-lived responses in PDAC patients treated as part of phase I/II CodeBreaK100 trial (NCT03600883). Among the 38 PDAC patients at a median follow-up of 16.8 months, ORR was 21%, DCR 84.2%, and mPFS of 3.98 months [58]. Preclinical synergy studies and understanding of the resistance mechanisms led the field of *KRAS G12C* inhibition to combination therapies such as EGFR inhibitors and/or immune checkpoint inhibitors (ICIs) [59] (Table 4).

While the drugs targeting *KRAS G12C* are more advanced in the field of drug development, *KRAS G12D* (~50%), *G12V* (~30%), and *G12R* (~10%) are the more common *KRAS* mutations in PDAC. Many studies support the notion that not all *KRAS* mutations are the same [60,61]. Understanding the similarities and differences can help design *KRAS* mutation-selective and pan-mutant inhibitors. Table 4 presents examples of mutant-specific and mutant nonspecific studies targeting *KRAS* mutations.

### 4.2. Targeting the Downstream Pathway of KRAS

The constitutive activation of the MAPK pathway is a hallmark of PDAC. Targeting MEK alone has not led to meaningful and/or durable responses in PDAC [62]. Preclinical studies suggested increased dependency of PDAC on autophagy in the context of MEK or ERK inhibition [63,64]. Furthermore, both MEK/ERK and autophagy inhibition are linked to the enhanced efficacy of ICIs in PDAC [65,66]. Multiple trials are evaluating the role of the combination of MEK or ERK inhibition with autophagy inhibition and/or ICIs (Table 4).

### 4.3. KRAS-Wild Type Tumors

*KRAS*-wild type tumors represent ~10% of PDAC tumors [67]. These tumors are rich in potentially actionable alterations. *BRAF* alterations are common in this population and can be targeted with BRAF, MEK, or BRAF plus MEK inhibitors [68]. NRG1 fusion, an alteration with a predilection for younger KRAS-Wild type patients [69], can be targeted by pan-ERBB inhibitors such as Afatinib. Fusions in genes such as *RET*, *ALK*, *ROS1*, *NTRK 1/2/3*, *MET*, *FGFR 1–3*, and mutations in *EGFR* are among the other alterations with available targeted therapies [67].

### 4.4. Somatic Mutations in the Homologous Recombination Repair (HRR) Pathway

Patients with somatic mutations in the HRR pathway genes leading to homologous recombination deficiency (HRD), specifically if biallelic (defined as pathogenic mutations in both alleles of the HRR gene or presence of a pathogenic mutation in one allele and loss of the wild-type allele), can also benefit from treatment with platinum-based chemotherapy [70]. Among the patients with PDAC, the prevalence of HRD varies depending on the testing method—14% to 16% with targeted next-generation sequencing and up to 24% to 44% with whole exome and genome sequencing [71]. Efforts are ongoing to standardize the definition of HRD in PDAC, expand access to tests that detect HRD [68], and find the best biomarker that can predict response to platinum, PARP inhibitors, and other DNA damaging agents (Table 4).

### 4.5. Cyclin-Dependent Kinase Inhibitor 2A (CDKN2A) Alterations

*CDKN2A* is a tumor suppressor gene located on chromosome 9p. *CDKN2A* alteration is frequent in PDAC. Nearly 29% of PDAC patients harbor *CDKN2A* deletions, and 21% harbor *CDKN2A* mutations [72]. Alterations in *CDKN2A/B* are associated with *CDK4/6* upregulation. Monotherapy with CDK4/6 inhibitor palbociclib did not result in meaningful responses in the TAPUR study [73]. However, the combination of CDK4/6 inhibitor plus MEK inhibitor has demonstrated preliminary activity in patients with concomitant *KRAS* and *CDKN2A/B* alterations [74].

The methylthioadenosine phosphorylase (*MTAP*) gene, encoding an enzyme crucial for methionine and adenine salvage, is in close proximity to the *CDKN2A* gene. Deletions of *CDKN2A* are therefore frequently associated with co-deletions of *MTAP*. MTAP loss results in the accumulation of methyladenosine, which in turn results in partial inhibition of the activity of the protein arginine methyltransferase 5 (PRMT5) [75]. MTAP deficient cells are susceptible to PRMT5 inhibitors or compounds that reduce the activity of PRMT5 protein, such as methionine adenosyltransferase 2a (MAT2A) inhibitors [76]. AG-270 is a MAT2A inhibitor in clinical trials in PDAC (Table 4).

## 5. Immunotherapy

The only form of mPDAC that responds well to immunotherapy with an immune checkpoint inhibitor (ICI) is the tumor harboring mismatch repair deficiency (dMMR), leading to high microsatellite instability (MSI-H). However, the MSI-H signature is rare in PDAC, occurring only in <1% of patients and most often associated with Lynch syndrome [77,78]. Robust responses to ICIs have been documented in MSI-H pancreatic cancer patients [79,80,81,82], with an ORR varying from 18.2% [82] to 77% [79]. Currently, pembrolizumab, a PD-1 blocker, is approved by the FDA for patients with chemotherapy-refractory advanced MSI-H/dMMR PDAC.

As most pancreatic tumors lack a MSI-H signature and the tumor microenvironment (TME) is heavily infiltrated by immunosuppressive cell populations, including tumor-associated macrophages(TAMs), regulatory T cells (Tregs), myeloid-derived suppressor cells (MDSCs), and neutrophils that enable cancer cells to evade the immune system, currently available immunotherapies have been proven to be ineffective in pancreatic cancer [83]. Several trials have confirmed that the activity of ICI monotherapy in mPDAC is disappointing. A phase II trial with 27 patients of mPDAC showed no objective responses to ipilimumab, an anti-cytotoxic T-lymphocyte-associated protein 4 (CTLA4) agent [84]. A randomized phase II study with durvalumab (anti-PD-L1 drug) with or without tremelimumab (anti-CTLA4 agent) in the second-line setting also reported a disappointing response-ORR of 3.1% with the combination therapy and 0% with the durvalumab monotherapy [85]. Conversely, treatment with a combination of ipilimumab and nivolumab in patients with Homologous Recombination Deficiency (HRD) Pathogenic Germline Variants has shown encouraging preliminary results. In a small retrospective series in mostly PDAC patients with HRD, treatment with ipilimumab/nivolumab combination resulted in an objective response rate of 42%, with a disease control rate of 58%. Four patients achieved CR without evidence of disease progression 11 to 41 months after the initiation of immunotherapy [86].

Several strategies are being investigated to recruit the immune system against pancreatic tumors. One of the promising strategies is targeting CD40 with agonist antibodies [87]. CD40, a cell-surface protein belonging to the tissue necrosis factor (TNF) superfamily and a critical component of both adaptive and innate immune systems, is expressed on a wide variety of immune cells, including macrophages, monocytes, dendritic cells, and B cells. CD40 agonist antibodies exploit the following mechanisms to activate anti-tumor immunity: (1) activation of antigen-presenting cells (APCs) resulting in the generation of tumor-specific cytotoxic T cells that do not require helper CD4 T cells, (2) direct stimulation of macrophages that depletes tumor stroma, converting ‘cold’ tumors into ‘hot’ tumors, and (3) APCs activated by CD40 stimulation produce interleukins (IL) IL-12 and IL-15 facilitating NK cell-mediated tumor cell killing.

The rationale for the clinical development of CD40 agonist antibodies as monotherapy or in combination with ICIs, vaccines, chemotherapy, and radiations has emerged from numerous preclinical studies in murine models [87]. A combination of CD40 agonist and gemcitabine in a small group of treatment-naïve mPDAC patients has reported promising activity- 88% response rate by FDG-PET, partial response (PR) in 4 out of 21 patients, and stable disease (SD) in 11 patients according to the RECIST criteria [88]. A study is currently undergoing to evaluate the safety and efficacy of CDX-1140 (CD40 agonist antibody) alone (Part 1) or in combination with CDX-301, a hematopoietic cytokine (Fms-related tyrosine kinase 3 ligand) (Part 2), pembrolizumab (Part 3), or chemotherapy (Part 4) in patients with a variety of solid tumors (NCT03329950).

The other emerging immunotherapy approach is vaccine therapy. GVAX pancreas, an irradiated allogeneic whole pancreatic tumor cell vaccine programmed to express granulocyte-macrophage colony-stimulating factor (GM-CSF), combined with cyclophosphamide and CRS-207, live-attenuated Listeria monocytogenes-expressing mesothelin, showed a median OS of 9.7 months in a cohort of 90 patients with previously treated mPDAC, a result seemingly better than historical OS achievable with chemotherapy [89]. However, a subsequent larger phase IIb study with this combination did not show improved OS compared to chemotherapy [90]. A randomized phase II trial in previously treated patients also failed to improve OS with the nivolumab and GVAX/CRS 207 combination [91]. A phase II randomized study is currently recruiting to evaluate the efficacy of GVAX/CRS 207 plus dual ICI (ipilimumab and nivolumab) combination (NCT03190265).

Chimeric antigen receptor (CAR)-T cell therapy that employs genetically-engineered T cells directed to specific cancer-associated antigens is also being evaluated in PDAC. Preliminary results of preclinical and phase I studies in PDAC are encouraging [92,93], and several phase I/II trials are currently underway with CAR-T cell therapy in a variety of solid tumors, including PDAC (NCT02744287, NCT03159819).

## 6. Therapies Targeting Tumor Microenvironment and Metabolomics

Deranged mitochondrial metabolism is a common characteristic in cancer cells [94]. Devimistat (CPI-613), a lipoate analog that can inactivate two crucial enzymes in the Krebs cycle, specifically in tumor cells, was evaluated in the phase 3 AVENGER 500 trial along with modified FOLFIRINOX, compared to FOLFIRINOX alone (NCT03504423). Based on the press release from the company, the mOS was 11.1 months in the devimistat + chemotherapy arm vs. 11.7 months in the chemotherapy arm (HR 0.95, *p* = 0.66).

Altered amino acid metabolism is another feature of cancer. Tumor cells rely on the external supply of non-essential amino acids such as asparagine to survive [95]. L-asparagine depletes the plasma arginine and leads to cancer cell death [96]. Eryaspase, RBC encapsulated L-asparaginase, was tested in phase 3 Trybeca-1 trial and did not improve OS in patients with mPDAC treated with eryaspase + chemotherapy compared to chemotherapy alone in the second-line setting [97]. ADI-PEG 20, a pegylated arginine deaminase, and GnP led to an ORR of 45% in a small study in treatment-naive PDAC patients [98].

## 7. Targeting Stroma

The dense desmoplastic stroma in PDAC is associated with decreased drug delivery to the cancer cells [99]. Approaches to deplete the stroma in PDAC have been unsuccessful so far. PEGPH20, a pegylated human hyaluronidase, in combination with FOLFIRINOX compared to FOLFIRINOX alone in a hyaluronic acid (HA)-high unselected population led to inferior outcomes [100]. Furthermore, In the phase 3 trial of HA-high PDAC patients, the addition of PEGPH20 to GnP did not lead to improved OS or PFS [101]. Similarly, pembrolizumab plus PEGPH20 did not improve PFS compared to historical data in the advanced treatment-refractory setting [102].

Current approaches mostly focus on stromal remodeling and normalization (Table 4). Pamrevlumab (FG-3019) is a monoclonal antibody targeting connective tissue growth factors. Preclinical studies showed decreased tumor growth, metastasis, and fibrosis with the use of pamrevlumab [103]. A phase 3 study is evaluating the role of this agent in mPDAC (Table 4). Vitamin D receptor (VDR) is highly expressed in PDAC stroma, including among cancer-associated fibroblasts (CAFs), which are promoters of PDAC growth and aggressiveness [104]. Vitamin D agonists can shift CAFs to a more quiescent and less aggressive form reducing tumor growth and improving chemotherapy delivery [105]. Paricalcitol, a VDR agonist, is being studied in multiple PDAC trials (Table 4). Vitamin A derivative all-trans retinoic acid, with an established role in promyelocytic leukemia management, is undergoing testing for its ability to reprogram the stroma in PDAC [106]. The efficacy of ATRA + GnP is being explored in a phase 2b study (Table 4).

## 8. Future Perspective and Conclusions

The current treatment of advanced pancreatic cancer is largely empiric. The genomic profiling technology unraveling the signaling pathways that support tumor growth and propagation has created precision treatment opportunities for many cancer types, including PDAC. Although a road to precision oncology has not been clearly mapped out yet, the recent discovery of several treatment-relevant predictive biomarkers has helped the precision therapy paradigm. However, tumor heterogeneity ‘in space and time’ in pancreatic cancer poses formidable challenges in finding predictive biomarkers that can reliably inform clinicians to choose effective treatment for a given patient at a given point in time. A wide variety of research tools have emerged attempting to solve these problems.

Research to personalize chemotherapy in patients with PDAC is rapidly evolving. Multiple approaches to personalize chemotherapy are currently ongoing. Based on tumor transcriptomics, distinct molecular subtypes of PDAC have been identified. Of these subtypes, the classical and basal-like patterns in a modified Moffitt classification have shown prognostic and predictive values [107,108]. The COMPASS trial that performed real-time RNA and whole-genome sequencing (WGS) in patients undergoing first-line chemotherapy identified improved chemotherapy response in the classical subtype, especially with FOLFIRINOX [107]. Furthermore, there was a suggestion of possible improved outcomes in the basal-like tumors treated with GnP [107]. More importantly, this study revealed that GATA6 RNA expression could potentially distinguish the two sub-types [107]. Based on these data, GATA 6 tissue immunohistochemistry (IHC) has been proposed as a simpler surrogate to differentiate between the two sub-types [109]. The PASS-01 trial is prospectively evaluating if the response to GnP and FOLFIRINOX correlates with GATA-6 expression in the first-line setting (NCT04469556). Alternatively, Purity Independent Subtyping of Tumors (purist) was developed as a single sample classifier that could successfully identify tumor subtypes with high performance even utilizing tissues with limited cellularities, such as those obtained by fine-needle aspiration at the time of diagnosis [110]. The basal-like tumor subtype based on purist classification system was associated with resistance to FOLFIRINOX [110]. The ongoing phase 2 PANCREAS trial is evaluating if the purist classification system can guide treatment in the neoadjuvant setting (NCT04683315).

Patient-derived organoids (PDOs) are another tool that can potentially provide a unique opportunity to develop timely models for treatment response prediction before such treatments are administered to the patient [111]. This approach, however, is currently limited by the pace of organoid development and the inability to replicate intratumor heterogeneity and tumor microenvironment. Circulating tumor cells (CTCs) may also be another tool to help in treatment response prediction [112]. The PASS-01 trial, a prospective randomized phase 2 trial evaluating GnP vs. modified FOLFIRINOX in the first-line setting, aims to systematically synthesize data from PDOs, WGS, RNA sequencing, and serial circulating tumor DNA, CTCs and help inform precision therapy choices (NCT04469556).

The universal availability of genomic profiling has provided the opportunity to understand the biology of tumors on a personal level. With the inevitable progress of the multi-omics testing of tumors and the ability for an in-depth evaluation of tumor characteristics through technologies such as single-cell RNA, a multidisciplinary molecular tumor board including clinical/translational/basic investigators, geneticists, bioinformatics, pharmacist, mediation acquisition specialist, and clinical trial navigators needs to be available to determine the choice of initial treatment and subsequent therapies based on a clonal evolution of the tumor. Such an approach has been previously implemented [113]. Ideally, this should be open to all treatment oncologists irrespective of the type of the treating institution (academic vs. community) and locally available resources. A successful example of such an approach is available (https://www.nature.com/natcancer, accessed on 15 April 2022). Utilizing such an approach, testing and optimizing combinatorial targeted therapies in PDAC is reachable. However, it is important to highlight that genomic profiling technology is not ubiquitously available in all parts of the world, which poses a barrier to accessing novel therapies. As technology matures and becomes cheaper, hopefully, it will be within reach of the majority of the world.

The liquid biopsy that encompasses one or more circulating biomarkers, including cell-free DNA (cfDNA), tumor-derived exosomes, and circulating tumor cells, has shown promise as a novel tool to supplement the care continuum for patients with mPDAC, although not yet universally adopted for routine clinical use [114]. The use of cfDNA-based tumor genomic profiling is particularly important in the setting of metastatic disease after progression on standard treatment. Preliminary data suggest that cfDNA has a higher concordance with metastatic lesions than primary tumor tissue [115]. cfDNA analysis may identify the actionable mutations, broadening treatment options in the setting where obtaining an additional tissue biopsy is challenging. However, prospective studies need to be conducted to assess if this theoretical advantage can be translated into a quantifiable clinical benefit.

In conclusion, the modest efficacy of chemotherapy in advanced PDAC has shifted the focus of current research from cytotoxic chemotherapy development to biomarker-driven precision therapy. The remarkable advancements in the understanding of molecular biology of PDAC, especially in the areas of DNA repair mechanisms, tumor microenvironment, and metabolomics, have brought new opportunities for novel therapy development. However, several important barriers have to be overcome before precision therapy can be successfully implemented, including intratumor heterogeneity, multiple signaling pathways supporting cancer growth, the signaling pathway redundancy of RAS, and clonal evolution. Both serial liquid biopsy and the multi-omic evaluation of tumors might serve as a new tool to determine the optimum treatment for each patient.

## Figures and Tables

**Table 1:** Select trials evaluating efficacy and safety of first-line chemotherapy in metastatic pancreatic cancer.

Regimen	Study Design	*n*	Median OS	ORR	Toxicity	Comments	Reference
Gemcitabine vs. 5-FU	Phase III	126	5.6 months vs. 4.4 months (*p* = 0.0025)	5.4% vs. 0%	Neutropenia ≥ grade 3: 25.9 % vs.4.9% (*p* < 0.001)	Survival beyond 12 months: 18% vs. 2%. Clinical benefit, the primary efficacy measure of the study, 23.8% vs. 4.8% (*p* = 0.0022).	Burris (1997) [10]
Gemcitabine plus cisplatin vs. gemcitabine	Phase III	195	7.5 months vs. 6.0 months (*p* = 0.15)	10.2% vs. 8.2%	Nausea and vomiting 22.2% vs. 5.9%; *p* = 0.0002	Median PFS 5.3 vs. 3.1 months (*p* = 0.053) Rate of SD 60.2% vs. 40.2%; *p* < 0.001	Heinemann (2006) [12]
Erlotinib plus gemcitabine vs. gemcitabine	Phase III	569	6.24 months vs. 5.91 months (*p* = 0.038)	8.6% vs. 8%	Higher frequencies of rash, diarrhea, infection, and stomatitis in the erlotinib + gemcitabine arm.	One-year survival was also greater with erlotinib plus gemcitabine (23% vs. 17%; *p* = 0.023)	Moore (2007) [15]
Gemcitabine + bolus 5-FU vs. Gemcitabine	Phase III	322	6.7 months vs. 5.4 months (*p* = 0.09).	3.4 months vs. 2.2 months (*p* = 0.02)	No significant differences between the two arms	ORR 6.9 % vs. 5.6%.	Berlin (2002) [9]
Gemcitabine plus capecitabine vs. gemcitabine	Phase III	533	7.1 months vs. 6.2 months (*p* = 0.08)	19.1% vs.12.4%	Grade ≥ 3 neutropenia 35% vs. 22%	The median PFS: 5.3 vs. 3.8 months (*p* = 0.004)	Cunningham (2009) [11]
FOLFIRINOX vs. gemcitabine	Phase III	342	11.1 months vs. 6.8 months (*p* < 0.001)	31.6% vs. 9.4%	Febrile neutropenia −5.4% in FOLFIRINOX group	The median PFS: 6.4 vs. 3.3 months (*p* < 0.001)	Conroy (2011) [7]
Nab-paclitaxel plus gemcitabine vs. gemcitabine	Phase III	861	8.5 months vs. 6.7 months (*p* <0.001)	23% vs. 7%	Grade ≥ 3 toxicities: neutropenia: 38% vs. 27%. Neuropathy: 17% vs. 1%.	The median PFS: 5.5 vs. 3.7 months (*p* < 0.001)	Von Hoff (2013) [8]
Nab-paclitaxel plus gemcitabine plus cisplatin	Phase 1b/2	25	16.4 months	71%	Grade ≥ 3 toxicities: Thrombocytopenia (68%), anemia (32%), and neutropenia (24%).	The median PFS was 10.1 months.	Jameson (2019) [16]
Gemcitabine, cisplatin plus veliparib vs. gemcitabine and cisplatin	Phase II	50	15.5 vs. 16.4 months (*p* = 0.6)	74.1 % vs. 65.2%	Grade 3 to 4 hematologic toxicities: neutropenia 48% vs. 30%. thrombocytopenia 55% vs. 9%	Median PFS: 10.1 vs. 9.7 months. The two-year OS rate for the entire cohort was 30.6% (95% CI, 17.8% to 44.4%).	O’Reilly (2020) [17]
NALIRIFOX (liposomal irinotecan + oxaliplatin + 5-FU/LV)	Phase I/II	56	12.6 months	34.4%	22 of 32 patients had grade ≥ 3 TRAEs, including neutropenia (31.3%), febrile neutropenia (12.5%) and hypokalemia (12.5%).	Median PFS 9.2 months.	Wainberg (2021) [18]

Abbreviations: OS, overall survival; ORR, overall response rate; 5-FU, 5-fluorouracil; PFS, progression-free survival; SD, stable disease; FOLFIRINOX, 5-FU, irinotecan and oxaliplatin; TRAE, treatment-related adverse events; LV, leucovorin.

**Table 2:** Published studies with maintenance treatment regimens in metastatic pancreatic ductal adenocarcinoma.

Study	Patient Population	*n*	Study Design	Induction Therapy	Maintenance Therapy	Outcome	Comments
POLO [31]	mPDAC with *BRCA1 or 2* germline mutation	154	Randomized phase III	At least 16 weeks of first-line platinum-based chemotherapy	Olaparib or placebo	mPFS 7.4 (olaparib) vs. 3.8 months (placebo) [HR, 0.53; 95% CI, 0.35–0.82; *p* = 0.004]	Most patients received induction FOLFIRINOX (79.3 % in the Olaparib group and 71% in the placebo group)
PACT-12 [36]	Unselected patients with mPDAC	56	Randomized Phase II	6 months of first-line chemotherapy	Arm A = Observation Arm B = sunitinib at 37.5 mg daily until progression or a maximum of 6 months	mPFS significantly longer with sunitinib maintenance (3.2 vs. 2 months, HR 0.51; 95% CI, 0.29–0. 89; *p* < 0.01	2-year OS was remarkably high with sunitinib (7.1% vs. 22.9%; *p* = 0.11)
Petrioli et al. [35]	Locally advanced or mPDAC >70 years old	36	Prospective observational study	3 cycles of gemcitabine and nab-paclitaxel	Gemcitabine	Six-month DCR- 61%, mPFS-6.4 months, and mOS-13.4 months.	
Chevalier et al. [33]	Unselected patients with mPDAC	321	Multicenter retrospective study	FOLFIRINOX (Median 9 cycles)	FOLFIRI-45%, 5-FU or capecitabine-35%, FOLFOX 17%	mOS- 16.1 months.	mOS and mPFS were similar between the groups receiving FOLFIRI or 5-FU
PANOPTIMOX-PRODIGE 35 [34]	Unselected patients with mPDAC	276	Randomized Phase II	6 months of FOLFIRINOX (arm A), 4 months of FOLFIRINOX followed by 5-FU/LV maintenance treatment (arm B), or a sequential treatment alternating gemcitabine and FOLFIRI every 2 months (arm C).		mOS: 10.1 months in arm A, 11.2 in arm B, and 7.3 in arm C.	Median survival without deterioration in quality-of-life scores was higher in the maintenance arm (B) at 11.4 months than in arms A (7.2 months) and C (7.5 months).

Abbreviations: mPDAC, metastatic pancreatic ductal adenocarcinoma; mPFS, median progression-free survival; HR, hazard ratio; mOS, median overall survival; DCR, disease control rate; 5-FU, 5-fluorouracil; LV, leucovorin; FOLFIRINOX, 5-FU, irinotecan, and oxaliplatin; FOLFIRI, 5-FU, and irinotecan; FOLFOX, 5-FU, and oxaliplatin.

**Table 3:** Studies with second-line chemotherapy in patients with metastatic pancreatic ductal adenocarcinoma.

Study	Study Design	*n*	Treatment Regimen	ORR (%)	Median PFS (Months)	Median OS (Months)	Additional Information
After progression on first-line FOLFIRINOX							
Portal et al. [39]	Prospective multicenter study	57	GA	18	5.1	8.8	Garde 3 or higher neurotoxicity 13%
Mita et al. [38]	Phase II	30	GA	13	3.8	7.6	70% of patients experienced grade 3 or 4 AEs
Viaud et al. [40]	Retrospective study	96	Gemcitabine	10	2.1	3.7	DCR 40%
After progression on first-line gemcitabine							
Wang-Gillam et al. [41]	Phase III	417	5-FU/LV/Nal-IRI vs. 5-FU/LV	17 vs. 1	3.1 vs. 1.5 (*p* < 0.001).	6.1 vs. 4.2 (*p* = 0.012)	Estimated one-year overall survival rate was 26% with nal-IRI + 5-FU/LV.
Oettle et al. [42]	Phase III	160	OFF vs. 5-FU/LV	-	2.9 vs. 2 (HR, 0.68; *p* = 0.019)	5.9 vs. 3.3 (*p* = 0.01)	Grade 1/2 neurotoxicity, 38.2% vs. 7.1%
Gill et al. [43]	Phase III	108	mFOLFOX6 vs. 5-FU/LV	13.2 vs. 8.5	3.1 vs. 2.9 (*p* = 0.99)	6.1 vs. 9.9 (*p* = 0.02)	Adverse events leading to study withdrawal- 20% vs. 2%
Kim et al. [44]	Phase II	39	mFOLFIRINOX	10.3	3.8	8.5	Grade 3–4 neutropenia occurred in 40% of patients
Sawada et al. [45]	Retrospective	104	mFOLFIRINOX	10.6	3.9	7	First-line regimen-GA.
Zaniboni et al. [46]	Phase II	50	FOLFIRI	8	3.2	5	6-mo survival rate-32%.
Xiong et al. [47]	Phase II	41	XELOX	2.5	2.5	6	The survival rate at 1 year was 21%.
Ettrich et al. [48]	Phase II	44	Docetaxel and oxaliplatin	15.9	1.8	10.1	DCR 47.7%

Abbreviations: ORR, overall response rate; PFS, progression-free survival; OS, overall survival; FOLFIRINOX, 5-FU/leucovorin/oxaliplatin/irinotecan;.GA, gemcitabine/abraxane; AE, adverse event; DCR, disease control rate; 5-FU, 5-fluorouracil; LV, leucovorin; Nal-IRI, nanoliposomal irinotecan; OFF, oxaliplatin/5-FU/leucovorin; HR, hazard ratio; m (modified)FOLFOX, 5-FU/leucovorin/oxaliplatin; mFOLFIRINOX, modified FOLFIRINOX; FOLFIRI, 5-FU/leucovorin/irinotecan; XELOX, capecitabine/oxaliplatin.

**Table 4:** Novel clinical trials with targeted therapy combinations.

Target Pathway/Gene	Study Agents with the Mechanism of Action	Study Phase	Clinical Trial Identifier
*KRAS G12C*	Adagrasib (KRAS G12C inhibitor) + Cetuximab (anti-EGFR agent)	1b	NCT03785249
	Adagrasib + BI-1701963 (SOS-1 inhibitor)	1/1b	NCT04975256 (KRYSTAL-14)
	Adagrasib + TNO155 (SHP-2 inhibitor)	1/2	NCT04330664 (KRYSTAL-2)
	Adagrasib Palbociclib (CDK 4/6 inhibitor)	1	NCT05178888 (KRYSTAL-16)
*KRAS G12D*	siG12D-LODER in combination with chemotherapy	2	NCT01676259 (PROTACT)
*Pan-KRAS*	BI-1701963 (SOS-1 inhibitor) +/− Trametinib (MEK inhibitor)	1	NCT04111458
	RMC-4630 (SHP-2 inhibitor) + Cobimetinib (MEK inhibitor)	1/2	NCT03989115
*KRAS*-based immunotherapy	V941 (KRAS-targeted vaccine) +/−Pembrolizumab	1	NCT03948763
	Poly-ICLC (a TLR3 Agonist) + nivolumab & ipilimumab	1	NCT04117087
	LY3214996 (ERK inhibitor) + Hydroxychloroquine (targets autophagy)	2	NCT04386057
	Trametinib Hydroxychloroquine	1	NCT04132505 (HOPE)
	Binimetinib Hydroxychloroquine	1	NCT03825289 (THREAD)
	Cobimetinib Hydroxychloroquine Atezolizumab	1/2	NCT04214418 (MEKiAUTO)
Targeting HRD	Niraparib Dostarlimab	2	NCT04493060
	Olaparib Pembrolizumab	2	NCT04666740 (POLAR)
	CX-5461 (RNA polymerase I transcription inhibitor)	1	NCT04890613
Targeting *CDKN2A* and *MTAP* deletions	AG-270 + GnP	1	NCT03435250
Targeting tumor metabolism	SBP-101 + GnP	1	NCT03412799
	SBP-101 + GnP	2/3	NCT05254171
	L-glutamine + GnP	1	NCT04634539
	GP-2250 + gemcitabine	1/2	NCT03854110
Targeting tumor stroma	Pamrevlumab (antibody against connective tissue growth factor) + GnP as first or second-line	2/3	NCT04229004 (Precision Promise)
	Paricalcitol (Vitamin D analog)	2	NCT04054362
	Paricalcitol Nivolumab	2	NCT02754726
	Paricalcitol hydroxychloroquine	2	NCT04524702
	Paricalcitol + 5-Fu/LV/Nal-IRI	1	NCT03883919
	+/− ATRA + GnP	2	NCT04241276
	Defactinib (FAK inhibitor) + Pembrolizumab	2	NCT03727880

Abbreviations: EGFR, epidermal growth factor receptor; HRD, homologous recombination deficiency; 5-FU/LV/Nal-IRI: 5-Fluorouracil/leucovorin/liposomal irinotecan; GnP, gemcitabine and nab-paclitaxel; ATRA: All-trans retinoic acid; FAK, focal adhesion kinase.
